# The Societal and Patient-Level Burden of Sickness Absenteeism in Patients with Rheumatoid Arthritis, Psoriatic Arthritis and Ankylosing Spondylitis Across Two Decades in Slovenia

**DOI:** 10.2478/sjph-2026-0015

**Published:** 2026-06-01

**Authors:** Mitja Oblak, Aleša Lotrič Dolinar, Žiga Rotar, Matija Tomšič, Petra Došenović Bonča

**Affiliations:** National Institute of Public Health, Trubarjeva cesta 2, 1000 Ljubljana, Slovenia; University of Ljubljana, School of Economics and Business, Kardeljeva ploščad 17, 1000 Ljubljana, Slovenia; University Medical Centre Ljubljana, Department of Rheumatology, Vodnikova cesta 62, 1000 Ljubljana, Slovenia; University of Ljubljana, Faculty of Medicine, Vrazov trg 2, 1000 Ljubljana, Slovenia

**Keywords:** Arthritis, Spondyloarthritis, Costs of illness, Indirect costs, Sickness absenteeism, artritis, spondiloartritis, strošek bolezni, posredni, stroški, absentizem

## Abstract

**Introduction:**

To analyse the long-term dynamics of the burden of sickness absenteeism (SA) at the societal and patient levels in employed patients with selected inflammatory rheumatic diseases (IRDs).

**Methods:**

The burden of SA was analysed over 2 decades prior to the COVID-19 pandemic, with a focus on the subperiod following the introduction of the rheumatology clinical registry biorx.si. Population data for full-time employees on sick leave due to rheumatoid arthritis (RA), psoriatic arthritis (PsA), and ankylosing spondylitis (AS) were obtained from the national administrative database. The societal burden of SA was defined as the total annual number of calendar days on sick leave. The patient-level burden of SA was defined as the average annual number of days on sick leave per patient which was further disaggregated into the average annual number of sick leave episodes per patient and the average annual number of days per sick leave episode. The costs of SA were estimated using the human capital approach. An exponential trend method was used for analysis, and time series were tested for structural breaks.

**Results:**

The societal burden of SA during the subperiod following the registry introduction decreased, on average, by 3.3% annually among RA patients. The average annual increase in the societal burden of SA was marginal for PsA patients (0.4%) and substantial for AS patients (8.7%). The societal and patient-level burdens of SA varied by sex and job sector. The patient-level burden of SA, however, decreased on average by 2.5% annually for RA patients and increased marginally for both PsA and AS patients. The key pattern suggesting potential improvements in the patient-level burden of SA for the analysed IRDs was an increase in the number of sick leave episodes per patient, offset by shorter episode duration.

**Conclusions:**

A study of the societal burden of SA, including its components, and the patient-level burden of SA can support the development of more effective strategies for managing SA and facilitating a faster return to work.

## Izvleček

**Uvod:**

Analizirati dolgoročno dinamiko bremena bolniške odsotnosti (BO) na družbeni in pacientovi ravni za zaposlene paciente z izbranimi vnetnimi revmatskimi boleznimi (VRB).

**Metode:**

Breme BO smo analizirali tekom dveh desetletij s poudarkom na podobdobju po uvedbi revmatološkega kliničnega registra biorx.si. Populacijske podatke za zaposlene s polnim delovnim časom na bolniškem staležu zaradi revmatoidnega artritisa (RA), psoriatičnega artritisa (PsA) in ankilozirajočega spondilitisa (AS) smo pridobili iz nacionalne administrativne zbirke podatkov. Izdatke BO smo ocenili z uporabo pristopa človeškega kapitala. Za analizo smo uporabili metodo eksponentnega trenda, časovne vrste pa smo testirali za strukturne prelome.

**Rezultati:**

Družbeno breme BO se je v obdobju po uvedbi registra v povprečju znižalo za 3,3 % letno za paciente z RA. Povprečno letno zvišanje družbenega bremena BO je bilo neznatno za paciente s PsA (0,4 %) in znatno za paciente z AS (8,7 %). Kljub temu se je breme BO na ravni pacienta v povprečju znižalo za 2,5 % letno za vse bolnike z RA in neznatno zvišalo za paciente s PsA in AS. Ključni vzorec, ki pojasnjuje spremembe pacientovega bremena BO za vse analizirane VRB, je naraščanje števila epizod bolniškega staleža, ki so se izravnale z njihovim krajšim trajanjem.

**Zaključki:**

Predlagan nov pristop k merjenju dinamike BO tako na družbeni kot pacientovi ravni zagotavlja dodatne informacije deležnikom iz zdravstva s ciljem, da ublažijo breme BO in olajšajo hitrejšo vrnitev na delo.

## 1 INTRODUCTION

Inflammatory rheumatic diseases (IRDs) result in a considerable economic burden to society, consisting of direct, indirect, and intangible costs (1). Research on the economic burden of major IRDs, comprising rheumatoid arthritis (RA), psoriatic arthritis (PsA) and ankylosing spondylitis (AS) (2), indicates that the costs of productivity losses, particularly those attributable to sickness absenteeism (SA), are substantial (3,4,5) and contribute significantly to the overall economic burden of IRDs (6,7,8,9). A study of German patients with IRDs, for example, reported as early as 2 decades ago that the costs of SA accounted for 11–17% of total annual costs of major IRDs (10). To mitigate the costs of SA and prevent progression from SA to work disability in patients with IRDs, it is important to first address both the magnitude and determinants of SA, which were found to vary between studies with different study populations, observed time periods, research designs, and SA indicators used (11). This is particularly relevant given a notable increase in the overall burden of SA at the European level over the past two decades (12).

Examining the long-term dynamics of SA may help identify its determinants and provide insights for healthcare stakeholders aiming to mitigate SA in patients with chronic conditions more effectively. A few studies (13,14,15,16) on SA burden in patients with IRDs using national administrative data reported a long-term decrease in the average annual sick leave duration per patient, potentially suggesting more favourable work outcomes for these patients. However, in addition to studying changes in the SA burden at the patient level, a greater focus on assessing the burden from a societal perspective that accounts for all affected stakeholders is also needed. This is particularly relevant for the management of SA burden in healthcare systems with a shared burden between patients and payers of sickness benefits, including public payers and employers. Studying contextual factors associated with SA is also important for designing effective strategies to improve work outcomes in patients with IRDs (17). While there is some evidence on sex-related differences in SA (18), less is known about SA determinants, such as the nature of work and workplace-related characteristics that were identified as relevant determinants of SA in patients with AS (17). Job sector characteristics are also important because varying employment rates across sectors may influence SA (19). SA and related work outcomes can also be influenced by external shocks, such as COVID-19 labour market policies (20), as well as technological advancements and improved access to biologic therapies, which are associated with better health outcomes for patients with IRDs (21,22,23).

This study investigates the long-term dynamics of SA among Slovenian patients with RA, PsA, and AS during 2001–2019. The first aim is to investigate changes in the societal burden of SA for the studied IRDs and the associated costs. The second aim is to study changes in the patient-level burden of SA, accounting for both the frequency and duration of sick leave episodes. Special attention is given to examining the dynamics of both SA burdens in the subperiod following the introduction of the clinical registry biorx.si aimed at improving access to biologic therapies. We also assess whether changes in SA burdens varied by sex and observe which patients are on sick leave according to key job sectors. Changes in employment are also observed for the latter, given that the number of those eligible for SA increases with higher employment rates.

## 2 METHODS

### 2.1 Study design and population

This observational study uses an anonymised dataset on sick leave episodes obtained from the national administrative database of the National Institute of Public Health of Slovenia (NIJZ). The patient-level dataset includes all sick leave episodes of full-time employees with RA, PsA, and AS, coded according to ICD-10 as the cause of SA, that occurred during 2001–2019 in Slovenia. Sick leave episodes are recorded at the time of their formal termination; thus, individual sick leave episodes longer than 365 days (366 days in leap years) can span consecutive calendar years. Sick leave episodes were hence split between relevant consecutive years to enable observation of the societal burden of SA at the annual level. Sick leave episodes without anonymised patient identifiers were excluded. After data clean-up, the final dataset comprised 17,570 sick leave episodes among 12,934 Slovenian patients with RA, PsA, or AS who had at least 1 recorded sick leave episode due to their diagnosed IRD. The dataset consists of patients on sick leave due to seropositive RA (M05) or other types of RA (M06), PsA (arthropathic psoriasis (L40.5) or psoriatic and enteropathic arthropathies (M07.0–M07.3)), and AS (M45).

### 2.2 Indicators of the burden of SA and other variables

Typical SA measures derived from, e.g., Eurostat, OECD, and WHO datasets include indicators such as the global sickness absence rate, the frequency rate, and the absolute crude absence rate (12). Such indicators, regularly reported in Slovenia by NIJZ (24), are also used to indicate the general burden of SA by identifying the share of the population on sick leave as a percentage of contracted working time. In this study, we use the NIJZ database to derive a set of 5 indicators to identify the societal burden of SA and break it down into several components, including the patient-level burden. The first indicator is the cumulative duration of sick leave, measured as the total annual number of calendar days on sick leave, to reflect the societal burden of SA. Two additional indicators, i.e., the total annual number of patients on sick leave and the average annual number of calendar days on sick leave per patient, are also studied, as they jointly determine the societal burden of SA. While the number of patients on sick leave is influenced by both the disease burden and employment rates, the average annual number of calendar days on sick leave per patient represents the patient-level burden of SA. The patient-level burden of SA can be further disaggregated into 2 indicators: the average annual number of sick leave episodes per patient and the average annual number of calendar days on sick leave per sick leave episode. Changes in the number of episodes and their duration indicate shifts in patient’s SA patterns over time.

Other variables used in the analysis are sex, job sector, and employment rates. Job sectors from the analysed dataset are defined according to the NACE Rev. 2 classification as the primary economic activity of a business or organisation (25), where the patient had been employed at the time of the recorded sick leave episode. Data on economic activities have been collected in Slovenia since 2002, and NACE Rev. 2 came into force in 2008; thus, any records before 2008 were recoded accordingly. The employment rate was defined as the proportion of employed persons to the total working-age population (26), and data were obtained from the publicly available database of the Statistical Office of the Republic of Slovenia (SURS) (27).

### 2.3 Statistical analysis

The long-term dynamics of SA for selected IRDs in Slovenia during the 2001–2019 period were analysed separately into 2 subperiods, based on the introduction of the biorx.si registry. biorx.si was introduced in 2008 for RA patients and in 2011 for PsA and AS patients (21). Exponential trend analysis was applied to derive average annual growth rates (measured in %). The Supremum Wald test was used to test for potential structural breaks in the entire 2001–2019 time series to provide additional evidence on whether the year of biorx. si introduction may have influenced changes in SA dynamics.

Total annual nominal costs of SA were estimated using the human capital approach (HCA) by multiplying the lost calendar days by the daily labour costs per worker during 2008–2019. Daily labour costs were obtained from the public SURS database, which has collected these records since 2008 (28). The real growth rates of SA costs were derived by applying the Consumer Price Index (CPI) published by SURS with 2008 as the base year. Linear trend analysis was used to determine the average annual absolute changes in costs. The long-term nominal cost dynamics and real growth rates were compared with the reference populations of a) SA due to diseases of the musculoskeletal system and connective tissue (M00–M99) and b) SA due to all ICD-10 diagnoses (A00–Z99). Data on the reference populations were obtained from the public NIJZ database of SA (24).

## 3 RESULTS

### 3.1 Study population characteristics

The study population comprised patients with selected IRDs aged 20–69 years who were full-time employees in businesses or organisations across diverse job sectors in Slovenia. Around two-thirds of the studied patients were employed in sectors C (manufacturing), G (wholesale and retail trade, repair of motor vehicles and motorcycles), O (public administration and defence, compulsory social security), P (education), and Q (human health and social work activities). Nearly half a million calendar days were lost due to sick leave of RA patients, which was more than 4 times higher compared to PsA or AS patients. RA patients diagnosed with M05 had the highest average annual number of days lost per patient due to sick leave (55.4 days/year) and the highest recurrence rate of sick leave per patient (1.41 sick leave episodes annually). On the other hand, the average yearly duration of sick leave per AS patient was 30.9% shorter than that of RA patients with M05, and the lowest sick leave recurrence was observed in RA patients with M06 and PsA patients, with an average of 1.31 episodes annually. Women represented 73.2% of RA patients; the numbers of men and women were similar in PsA patients; and men represented 61.8% of AS patients. Details on the study population’s characteristics are available in the accompanying research data repository (29).

### 3.2 The societal burden of SA

**Figure 1.**
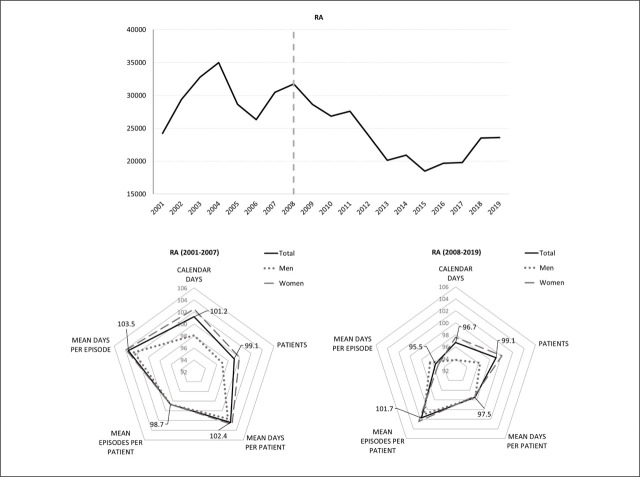
Dynamics of the total annual number of calendar days on sick leave during 2001–2019 and the average annual growth rates for indicators of SA in RA patients before and after the introduction of the biorx.si registry.

**Figure 2.**
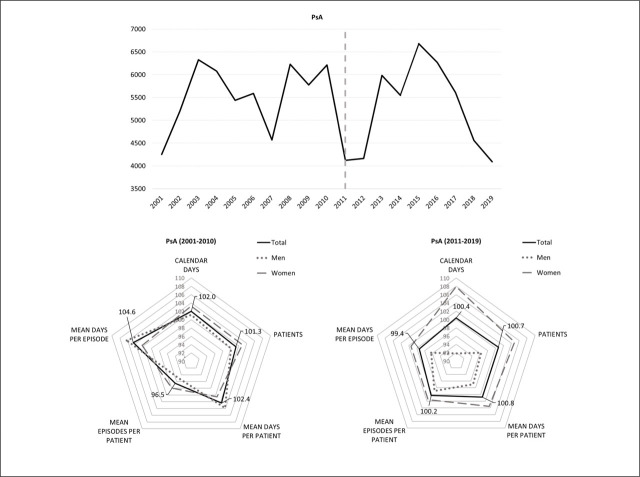
Dynamics of the total annual number of calendar days on sick leave during 2001–2019 and the average annual growth rates for indicators of SA in PsA patients before and after the introduction of the biorx.si registry.

**Figure 3.**
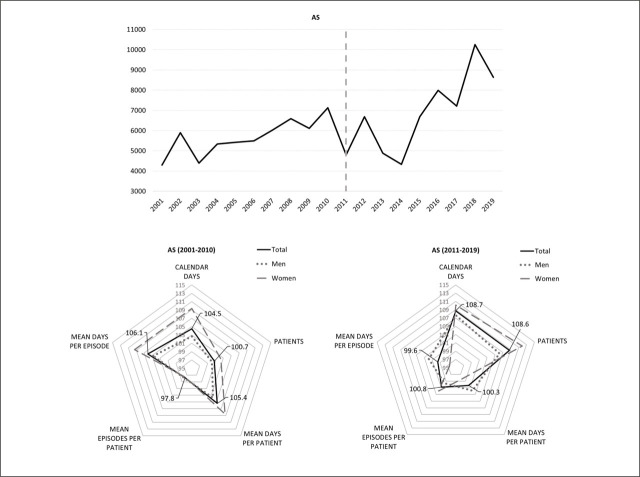
Dynamics of the total annual number of calendar days on sick leave during 2001–2019 and the average annual growth rates for indicators of SA in AS patients before and after the introduction of the biorx.si registry.

The societal burden of SA for RA patients, measured by the total annual number of calendar days on sick leave, decreased by 3.3% annually on average after the introduction of the biorx.si registry in 2008 (lower right panel of Figure 1), which was contrary to the average annual growth rate of 1.2% in the subperiod before the introduction of the biorx.si registry (lower left panel of Figure 1). This decrease in the societal burden of SA corresponded to a 1.5% average annual decrease in the costs of SA in real terms (−19,452 EUR/year in absolute terms), while the costs of SA for reference groups of diagnoses M00–M99 and A00–Z99 increased substantially after 2008 by real growth rates of 4.1% and 2.6%, respectively. The mean annual nominal costs of SA for patients with RA in the 2008–2019 period amounted to EUR 1,465,286, i.e. 2,674 EUR/year per patient (all results discussed in this section that are not shown in Table 1 and Figures 1–3 are available in the accompanying research data repository (29)).

**Table 1.** Average annual growth rates of SA indicators for RA, PsA and AS by sex during two subperiods.

**AVERAGE ANNUAL GROWTH RATES (%)**		**Calendar days**	**Patients**	**Mean days per patient**	**Mean episodes per patient**	**Mean days per episode**
**2001–2007**						
RA						
	Total	1.199	−0.938	2.394	−1.317	3.505
	Men	−1.894	−3.048	1.643	−1.322	2.537
	Women	2.485	−0.049	2.790	−1.313	3.889
M05						
	Total	2.735	1.741	1.617	1.744	0.741
	Men	4.160	2.731	1.613	4.707	−3.271
	Women	1.725	1.376	1.247	−1.218	1.574
M06						
	Total	0.534	−1.997	2.917	**−2.458**	**4.722**
	Men	−4.746	**−5.117**	1.114	**−3.457**	3.922
	Women	2.811	−0.607	3.718	**−1.458**	**4.958**
**2008–2019**						
RA						
	Total	−3.294	−0.907	**−2.527**	1.723	**−4.461**
	Men	−6.184	−3.729	−2.469	0.984	−3.503
	Women	**−2.416**	0.121	**−2.743**	**2.462**	**−4.905**
M05						
	Total	−0.049	**3.714**	**−3.926**	2.276	**−6.162**
	Men	−4.474	−0.312	−3.805	1.644	−5.737
	Women	1.527	**4.944**	**−3.800**	**2.909**	**−6.030**
M06						
	Total	**−5.585**	**−3.647**	−1.999	1.022	−3.316
	Men	−7.427	**−5.538**	−2.053	0.413	−2.404
	Women	**−5.178**	**−2.853**	−2.349	**1.631**	**−3.973**
**2001–2010**						
PsA						
	Total	1.980	1.273	2.397	−3.494	4.600
	Men	1.179	0.037	3.824	**−4.940**	6.263
	Women	3.256	2.912	0.554	−2.050	2.410
AS						
	Total	**4.476**	0.704	**5.395**	−2.218	**6.064**
	Men	2.654	0.067	3.742	−2.065	4.726
	Women	9.351	2.306	8.599	**−2.372**	9.452
**2011–2019**						
PsA						
	Total	0.397	0.729	0.770	0.247	−0.628
	Men	−8.155	−3.651	−3.055	−1.072	−3.648
	Women	7.963	**4.798**	3.527	1.565	1.420
AS						
	Total	**8.725**	**8.637**	0.270	0.786	−0.426
	Men	7.662	**6.159**	1.867	−0.395	1.817
	Women	**10.094**	**11.932**	−1.881	1.967	−3.462

Note: Exponential trend regression coefficients in bold font have R^2^ > 0.50.

The decreased societal burden of SA for RA patients after 2008 was observed together with the 2.5% average annual decrease in the burden of SA at the patient level measured by the mean annual number of days on sick leave per patient (similarly for men and women) and only a minor (0.9%) average annual decrease in the number of RA patients on sick leave. Although the number of women with subtype M05 on sick leave increased by 4.9%, this was offset by a minor decrease in the number of men with M05 and a decrease in the number of M06 patients of both sexes on sick leave (Table 1). Considerable average annual growth rates of 10.7%, 5.0% and 10.4% in the number of patients on sick leave were found in those M05 patients working in job sectors O, P, and Q, respectively, but not in job sectors C or G. While the number of employed persons in Slovenia during 2008–2019 increased on average by 0.2%, 1.9%, and 2.1% annually for women working in job sectors O, P, and Q, it increased by 1.4% and 2.6% for men working in job sectors P and Q, and decreased by 0.9% annually for men working in job sector O.

Although the societal burden of SA for PsA patients after the introduction of the biorx.si registry in 2011 increased on average by 0.4% annually, this increase was five times smaller compared to the prior subperiod (Figure 2). The corresponding costs of SA that amounted to EUR 336,347 per year on average decreased during 2008–2019 by 2.6% annually on average in real terms (−2,034 EUR/year), and the average annual cost per patient (2,890 EUR/year) was the highest among the 3 selected IRDs. The patient-level burden of SA and the number of PsA patients on sick leave increased slightly (< 1%) after 2011, when both indicators increased for women by 3.5% and 4.8% annually, respectively, and decreased for men (Table 1). The highest average annual increases of 5.0%, 14.2%, and 3.6% in the number of PsA patients were observed in job sectors O, P, and Q, but not job sectors C or G.

The societal burden of SA for AS patients increased by 8.7% annually on average after the introduction of the biorx.si registry, which was nearly twice as high as in 2001–2010 (Figure 3). The costs of SA also increased during 2008–2019 by an average of 3.7% annually in real terms (26,016 EUR/year). The average annual cost of SA for patients with AS was EUR 425,502, and the average cost of EUR 2,148/year per AS patient was the lowest among the selected IRDs. There was a minor increase in the patient-level burden of SA, with a 1.9% average annual increase in the average number of days on sick leave for men, offset by a similar decrease for women (Table 1). A notable (8.6%) average annual increase in the number of AS patients on sick leave, however, was observed for both sexes. Considerable average annual growth rates in the number of patients after 2011 were observed for AS patients working across all 5 selected job sectors (> 7%).

### 3.3 The patient-level burden of SA

A decreased patient-level burden of SA in RA patients after the introduction of the biorx.si registry was observed, together with a 1.7% average annual increase in the frequency of sick leave episodes per patient, offset by a −4.5% decrease in the average annual duration of these episodes. This general pattern was observed for men and women with both RA subtypes (Table 1). Structural break estimations suggest a change in the dynamics of the mean annual number of sick leave episodes per RA patient in 2008.

A minor increase (< 1%) in the patient-level burden of SA for patients with PsA (Figure 2) and patients with AS (Figure 3) after 2011 was observed. No associated structural break in the dynamics of the general pattern was estimated in the 2011. Contrary to RA, patient-level SA burdens and their components differed by sex among patients with PsA and AS. For men with PsA, both the frequency and duration of episodes declined, whereas the opposite was observed in women. For men with AS, the patient-level SA burden and duration of sick leave episodes increased, but the frequency of episodes decreased. For women with AS, the opposite pattern was observed compared with men (Table 1).

## 4 DISCUSSION

The general burden of SA is typically measured by the global sickness absence rate, the frequency rate, and the absolute crude absence rate (12). Our study shows that combining 5 indicators of SA can distinguish between the societal and patient-level burdens of SA and its components.

The societal burden of SA for RA patients decreased after 2008, mostly due to a decrease in the patient-level burden of SA, with similar dynamics observed in previous studies for RA patients (13,14,15), and a minor decrease in the number of patients on sick leave. Analysis of the societal burden of SA for RA patients by sex and RA subtypes revealed that the societal burden of SA decreased due to lower patient-level burden and a lower number of patients on sick leave for M06, regardless of sex, and for men with M05. On the other hand, the decreased patient-level burden for women with M05 was offset by the increasing number of women with M05. While the increasing number of women with M05 on sick leave could indicate an increasing burden of SA of this illness for women, our results reveal that the number of patients with M05 increased mostly in job sectors O, P, and Q. Given that the number of employed women in these job sectors increased in the observed period, the increasing number of women with M05 on sick leave may be partly attributed to increasing employment rates (19). As organisations from job sectors O, P, and Q are mostly in the public sector with higher job security and there is evidence suggesting women may self-select employment in the public sector for this reason (30, 31), more research is needed in the future to gain better insights into differences in SA between job sectors in Slovenia. Our results for RA patients also reveal that the decline in the patient-level burden of SA due to M06 was lower than that of patients with M05. Given that patients with M05 are reported to have worse clinical outcomes than those with M06 (32), further research is needed to address this finding. Nonetheless, our results suggest a decreasing societal burden of SA for Slovenian patients with RA.

A minor increase in the societal burden of SA for PsA patients after 2011 was driven by minor increases in both the number of patients and the patient-level burden, keeping the overall burden of SA relatively stable. Comparison between men and women with PsA, however, reveals that the societal burden of SA decreased for men due to lower patient-level burden and fewer men on sick leave, whereas the opposite was observed with the increasing societal burden of SA for women. This finding differs from a previous study, which estimated the decline in the number of days on sick leave during the calendar year following diagnosis for both sexes of PsA patients (18). Our results seem consistent with a previous finding for PsA patients that women have worse disease manifestations compared to men (33). While the results suggest a favourable declining trend in the societal SA burden of PsA for men, they also highlight the need to mitigate this burden for women with PsA more effectively. While the increasing employment levels in key observed job sectors can influence the number of women with PsA on sick leave, their increasing patient-level burden of SA warrants closer attention.

The increased societal burden of SA for AS patients after 2011 can be attributed to the growing number of patients on sick leave while keeping the patient-level burden relatively stable. The largest increase in the number of AS patients on sick leave was observed in job sector Q, which was also characterised by increasing employment levels for both sexes. While this may account for an increasing number of patients with AS on sick leave for both sexes, the increase in the patient-level SA burden for men was offset by its decrease for women. This result for the patient-level burden of SA is contrasted with findings from a previous study of patients with AS (18) and indicates that the increasing patient-level burden for men with AS requires further research.

Although we could not measure the causal effects of the introduction of the biorx.si registry on the SA burden, we do observe a decreasing time spent on sick leave annually as the main potential improvement for RA patients after 2008 compared to the prior subperiod. Minor increases in patient-level burden of SA observed for PsA and AS patients after 2011 were more than 3 times and nearly 20 times smaller than in the prior subperiod, respectively. While the patient-level burden of SA after the introduction of the biorx.si registry differed between analysed IRDs, the key pattern potentially explaining the patient-level burden of SA for all analysed IRDs was the increasing recurrence of sick leave episodes per patient that was offset by their shorter duration. The patient-level burden of SA before the introduction of the biorx.si registry was, however, observed for all analysed IRDs by decreasing recurrence of sick leave episodes per patient and by a longer duration of these episodes. Given that longer sick leave episodes have been shown to negatively affect return to work (34), our results may suggest favourable changes in the SA burden from the perspective of patients. Nevertheless, explaining changes in SA outcomes from the introduction of a clinical registry is a complex issue that cannot be adequately addressed within our study design. Current evidence suggests that the use of modern therapies has been associated with improvements in SA outcomes in patients with IRDs (35). Treatment with biologic therapies was shown to reduce the severity and duration of higher disease activity in patients with IRDs while not fully relieving disease flares (36), which may result in shorter but more frequent sick leave episodes as they resolve more quickly. Clinical registries can also contribute to improved disease control and patient monitoring (22, 23). Another possibility is that the observed decrease in the patient-level burden of SA may stem from shifts in the cost structure away from SA and toward other cost categories, such as presenteeism (37).

While the use of administrative national data at the patient level has been recognised for its potential to enhance evidence-informed decision-making in healthcare (38, 39), its application in our study has some limitations. Data included only full-time employees, and the results do not apply to patients in other types of working relationships. The observed time period in our data ended in 2019, not later, due to the disruption caused by the COVID-19 pandemic and the policy measures implemented, such as temporary layoff schemes with income compensation, which notably impacted SA statistics. Our study is also among the first to analyse SA among patients with IRDs by applying the NACE Rev. 2 classification of economic activities to define the job sectors of patients on sick leave. Because NACE Rev. 2 reports only the primary registered economic activity of businesses and organisations, it does not provide information about the type of job the patient performs (e.g. manual or sedentary work).

## 5 CONCLUSIONS

The total societal costs of SA for the three selected IRDs in Slovenia amounted to almost EUR 27 million over the 2008–2019 period. This paper shows that after the introduction of the biorx.si registry, there was a decrease in the societal burden of SA for RA for both sexes. With the exception of women with seropositive RA, both the number of patients on sick leave and the patient-level burden of SA decreased. While the societal burden of SA increased for patients of both sexes with AS, this was mostly due to the increased number of patients on sick leave. For women with AS, the growth of the societal burden of SA was decelerated through the lower patient-level burden of SA. For men with PsA both the number of patients on sick leave and the patient-level burden of SA decreased, while the opposite was observed for women. The key pattern suggesting potential improvements in the patient-level burden of SA for patients with the analysed IRDs after the introduction of the biorx.si clinical registry consisted of more frequent sick leave episodes offset by their shorter duration. While our findings are not causal and warrant further research, the indicators used to identify the societal burden of SA and to break it down into several components, including the patient-level burden, can support the development of more effective strategies for managing SA.
